# Examination of Breast Cancer Screening Knowledge, Attitudes, and Beliefs among Syrian Refugee Women in a Western Canadian Province

**DOI:** 10.1177/08445621211013200

**Published:** 2021-05-26

**Authors:** Louise Racine, Isil Andsoy, Sithokozile Maposa, Hassanali Vatanparast, Susan Fowler-Kerry

**Affiliations:** 1 College of Nursing, University of Saskatchewan, Saskatoon, Canada; 2 Department of Nursing, Karabuk University, Karabuk, Turkey; 3 College of Nursing, University of Saskatchewan, Prince Albert, Canada; 4 College of Pharmacy and Nutrition, University of Saskatchewan, Saskatoon, Canada

**Keywords:** Breast cancer screening, health beliefs model, breast cancer self-examination, mammograms, Syrian refugee women

## Abstract

**Background:**

Women living in the Arab world present low breast cancer screening rates, delayed diagnosis, and higher mortality rates.

**Purpose:**

To further explore the Muslim Syrian refugee women’s breast self-examination (BSE), utilization of clinical breast examination (CBE) and mammography.

**Methods:**

A cross-sectional descriptive exploratory study design was used. The sample consisted of 75 refugee women. Data were collected using Champion’s Health Belief Model Scale, the Cancer Stigma Scale, and the Arab Culture-Specific Barriers to Breast Cancer Questionnaire. Descriptive, Pearson correlation and logistic regression analyses were used to analyze the data.

**Results:**

A minority of women had BSE (32%), CBE (12%) and mammograms (6.7%) anytime during their lifetime. Women’s breast cancer screening (BCS) knowledge ranked at a medium level (M = 10.57, SD = 0.40). Low knowledge score, BSE information, policy opposition, responsibility, barriers to BSE, and seriousness were found to be statistically significant in women’s BSE practice. BSE benefits and religious beliefs significantly predict CBE Age, education, knowledge, responsibility, susceptibility, social barriers, and religious beliefs were statistically significant in women’s mammography use (p < .01).

**Conclusions:**

Participants’ breast cancer screening practices were low. Health beliefs, Arab culture and stigma about cancer affected women’s BCS practices. Faith-based interventions may improve knowledge and practices.

## Background and purpose

Cancer remains the second leading cause of death globally, with breast cancer the most common cause of cancer-related mortality (World Health Organization, [WHO], 2020). The WHO (2020) reports breast cancer’s incidence rate of 11.6% and a mortality rate of 6.6% among women worldwide. Although breast cancer appears as a disease of the developed world, more than 50% of the cases and 58% of the deaths occur in low- and middle-income countries (Azubuike et al., 2018). In these countries, inadequate health care systems and infrastructures represent significant structural barriers limiting access to breast screening methods (Baron-Epel et al., 2009; Elobaid et al., 2016; Sangaramoorthy & Guevara, 2017).

Early detection through cancer screening plays a significant role in reducing the morbidity and mortality of breast cancer. According to the WHO (2020), mammography is the most used screening method. Breast self and clinical breast examinations also increase breast cancer screening (Canadian Task Force on Preventive Health Care, 2011). Nevertheless, inequities in detecting breast cancer persist as screening rates are lower among non-Western refugee women in Canada (Ginsburg et al., 2015; Lofters et al., 2019; Vahabi, 2011; Vahabi et al., 2015; Woodgate et al., 2017).

Refugee populations present specific health and social vulnerabilities when accessing Western countries’ health care systems (Andreeva & Pokhrel, 2013; Saadi et al., 2012, 2015; Vahabi, 2011; Vahabi et al., 2015, 2016). For instance, U.S. studies show that Bosnian (Percac-Lima et al., 2012) and Iraqi refugee women (Saadi et al., 2012; Salman & Resick, 2015) may be more vulnerable to adverse health outcomes as they cope with pre-migration experiences of violence, warfare, post-traumatic stress disorder, malnutrition, and lack of health care in their home countries or neighboring countries’ refugee camps. Canadian and international researchers report that Iraqi and Syrian refugees were exposed to psychological and sexual violence and precarious living and housing conditions, which increase anxiety, stress, and low quality of life among these refugees (Al-Smadi et al., 2017a; Pottie et al., 2015). Leishmaniasis, measles, insomnia, anxiety, stress, hepatitis, typhoid, pulmonary tuberculosis, diabetes type 2 or hypertension are reported among refugee populations globally (Ekmekci, 2016; El-Khatib et al., 2013; Gammouh et al., 2015; Guruge et al., 2018). Refugees present a higher prevalence of chronic illnesses due to lack of appropriate medical care or lack of access to primary care health care, which adds to the burden of communicable diseases (Al-Smadi et al., 2017a; Gammounh et al., 2015; Pottie et al., 2015).

The loss of social capital post-migration negatively influences refugee health outcomes. Discrimination, lack of fluency, lack of knowledge of the host country’s health care system, loss of extended family and social network, and unemployment may contribute to refugees’ social isolation accentuating stress, anxiety, and depression (Al-Smadi et al., 2017b; McKeary & Newbold, 2010). A study conducted among Iraqi refugees in Jordan shows that gender, level of education, large families, and employment status significantly enhance positive coping strategies and may affect refugee women more than men as women tend to focus on caring for their children rather than seeking medical care (Al-Smadi et al., 2017b). Similarly, Canadian studies showed that refugees focus on learning the home country’s language and finding employment relegate health issues to the back burner as refugee men and women focus on immediate materials needs than seeking preventative health care services (Edge & Newbold, 2013; McKeary & Newbold, 2010; Wilkinson et al., 2017).

Evidence indicates that women from ethnocultural minority groups have a higher prevalence of advanced breast cancer, a lower 5-year survival rate, and a higher rate of breast cancer mortality than mainstream Canadian women (Vahabi, 2011; Vahabi et al., 2015, 2016). Women from Arabic-speaking countries appear to have the lowest breast cancer screening rates among ethnocultural groups living in Western countries (Hashim et al., 2018; Kwok et al., 2016; Petro-Nustas et al., 2012; Vahabi, 2011). Low survival rates occur in Muslim countries, for instance, Libya and Malaysia (Coleman, 2017) and Qatar (Donnelly & Hwang, 2013) due to delayed diagnosis.

The number of refugees fleeing to Canada and other receiving Western countries increases as the Middle East and Sub-Saharan countries remain involved in protracted civil and armed conflicts (Altunkaynak, 2016). In 2016, Western Europe received more than 1 million Middle Eastern and North African refugees. As of November 2016, 40,081 Syrian refugees migrated to Canada (Government of Canada, 2020). Syrian refugees in Canada are young, have lower levels of education, present a lack of English or French fluency, and have families of three or more children. Fifty-six percent of Syrian refugees to Canada are under the age of 14, and 87% of them are Islamic (Houle, 2019). The resettlement of such a high number of refugees is likely to impact Western countries’ healthcare systems (Boer, 2004). Massive migration of refugees from the Middle East and African countries is expected to continue in the coming years due to the collapse or destruction of infrastructures and economic systems in these countries (Houle).

An examination of the utilization of breast cancer preventative services in Muslim countries helps to understand breast cancer screening challenges among women. A survey of 22 Arab countries reveals that Lebanon, Bahrain, and Morocco have the highest incidence rate of breast cancer while Egypt, Morocco and Iraq have the highest mortality rates (Hashim et al., 2018). The authors attribute these discrepancies to the diversity among Arab countries in terms of economic development, health education, health care systems, and education and literacy level (Hashim et al., 2018). Some regions (Iraq, Syria) are affected by internal conflicts creating hurdles to early diagnosis and treatment (Hashim et al., 2018). Yet, the Arab world witnessed an increase in the rate of breast cancer (Hashim et al., 2018), low quality of life among women (Haddou Rahou et al., 2016) and low participation in breast screening activities (Donnelly et al., 2013a, 2013b). Epidemiological data indicate a constant rise in the incidence of breast cancer among Arab women (Hashim et al., 2018), however, this increased incidence can reflect better screening practices in high sociodemographic index (SDI) countries like the Gulf States compared to middle- and low-income countries in the rest of the Arab world.

Additionally, more than half of the Middle-East women are diagnosed with lymph nodes’ invasion revealing a more advanced stage of the illness and translating into higher mortality rates (Ghoncheh et al., 2015). In the Arab world, a higher proportion of women are diagnosed with breast cancer at a younger age compared to women in western Europe (Hashim et al., 2018). For example, 50% of Lebanese women presented with breast cancer at a mean age of 49.8 years compared to a proportion of 23% among U.S. women of the same age (Hashim et al., 2018). Breast cancer among younger women is more aggressive and fatal (Canadian Cancer Society, 2020), yet early diagnosis and screening increase the survival rate by 80% (Sun et al., 2017; WHO, 2020). In summary, empirical data demonstrate that breast cancer’s incidence rate among Muslim women has increased over the past 24 years, indicating an alarming trend in the Arab world (Hashim et al., 2018).

Cultural beliefs, attitudes, and knowledge may affect Muslim refugee women’s breast cancer screening (Alatrash, 2020; Chaka et al., 2018; Cohen & Azaiza, 2008). These low screening rates among Arab women may be associated with a lack of perceived needs to undergo regular health check-ups as women are not aware of breast cancer signs and symptoms before they are quite visible. Researchers report that most Egyptian women felt no need to consult a physician unless they were ill (Mamdouh et al., 2014). Cultural beliefs can shape cancer and breast cancer perspectives and influence lifelong health promotion and cancer preventative strategies (Jafari-Mianaei et al., 2017). Islamic faith may play a pivotal role in religious Muslim women’s lives as beliefs may influence perceptions around stigma and affect women’s willingness to participate in breast cancer screening practices and health-promoting behaviors (Hatefnia et al., 2010; Islam et al., 2017; Padela et al., 2015, 2018; Zorogastua et al., 2017). Women may fear losing social status related to their roles as wives and mothers (Cohen & Azaiza, 2008). Religious beliefs such as fatalism rooted in the view that cancer reflects God’s will may hinder access and utilization of breast cancer preventive strategies (Alatrash; Cohen & Azaiza, 2008). Some studies specifically explored perceived physical, cultural, social, and psychological barriers that may discourage Arab women from participating in breast cancer screening programs (Saadi et al., 2012, 2015). The fear of being diagnosed with a life-threatening disease like cancer, stigma, modesty, lack of familiarity or uneasiness with the breasts, low education level, and lack of health literacy translating into unawareness of the benefits of screening self-care activities represent barriers to breast screening (Alatrash; Chaka et al., 2018; Donnelly et al., 2013a, 2013b; Islam et al., 2017; Salman, 2012; Soskolne et al., 2007; Zorogastua et al., 2017).

Because of the risks inherent to breast cancer among women living in the Arab world and the increased migration rate to Western countries, Muslim refugee women represent a population of interest in a host country like Canada. As described earlier, cultural beliefs influence breast cancer preventative practices (Alatrash, 2020), yet little knowledge exists on how Muslim refugee women negotiate breast cancer screening practice in refugee receiving countries. Compared to other refugee women groups (Bedi & Devins, 2016; Lee et al., 2016; Poonawalla et al., 2014; Woodgate et al., 2017), knowledge about breast cancer screening practices and behaviours among Arab refugee women to Western countries remains sparse (Ahmed et al., 2015; Racine & Lu, 2015; Vahabi et al., 2015, 2016). Epidemiological data about breast cancer in Arab countries coupled with pre-and post-migration issues translate the urgency to explore refugee Muslim women’s behaviors, knowledge, barriers, and facilitators about breast cancer screening. There is a pressing need to understand the cultural, social, and gendered barriers that may affect the access and utilization of preventative breast cancer care among Muslim refugee women who recently resettled in a Western country like Canada. This study provides broader insights into Syrian refugee women’s breast health practices. We also aimed to explain which barriers and facilitators influence their participation in breast cancer screening preventative activities lie breast self-examination (BSE), clinical breast examination (CBE), and mammography.

## Methods and procedures

We used the Health Belief Model to guide the study. The Health Belief Model (HBM) is one of the first models adapted from psychology and behavioral sciences and applied to solve health problems (Champion, 1984). The Health Belief Model proposes that personal beliefs and perceptions about a disease influence health behaviors (Glanz et al., 2008). Perceived susceptibility to the illness, perceived seriousness, perceived benefits, and barriers to the behavior represent the theory's major concepts. The Champion’s Health Belief Model Scale (CHBMS) has been refined and revised in 1999 and adapted to ethnic, language, and cultural diversity. Champion (1984) defines perceived susceptibility as the subjective risks of presenting a condition or a disease. Perceived seriousness represents the perceived degree of personal threat a person has toward a specific illness or condition. Perceived benefits focus on the effectiveness or the positive effects of adopting a health-promoting behavior to avoid the disease. Perceived barriers refer to the negative effects related to adopting health-promoting behaviors. Finally, motivation relates to a positive appraisal of health and focuses on people’s willingness to achieve wellness and prevent illness by changing behaviors despite perceived barriers.

A cross-sectional descriptive exploratory study design was used to explore the barriers and facilitators that may influence Syrian refugee women’s participation in breast self-examination (BSE), clinical breast examination (CBE), and mammograms. The convenience sample consisted of 75 refugee women from two immigrant settlement agencies in a western Canadian province. The inclusion criteria were: 1) Having migrated to Canada within the past five years, 2) being a refugee, 3) being older than 18 years, 4) having no history of active or diagnosed breast cancer, 5) being self-identified being of Muslim faith, 6) having a basic level of literacy like being fluent in English or Arabic, (7) not having been diagnosed with breast cancer, and 8) willing to participate in the study.

## Data collection

 Ethical approval from the University of Saskatchewan Behavioural Ethics Board (REF 2018-BEH 55) was obtained before data collection. Data collection was conducted from July to December 2018. Surveys, consent forms, letters of information for participants, letters of information for their male relatives and recruitment advertisements were available in English and Arabic. Informed consent was obtained from each participant. Issues of confidentiality were attended by assigning a random number to each participant. Participants were informed that their participation was voluntary and that they had the right to withdraw from the study. A bilingual speaker translated the English materials into Arabic and checked the translations for accuracy. Surveys were administered at two immigrant settlement agencies. Data were collected using an Arab-speaking interpreter in real-time interviews, and participants with reading difficulties received assistance from the interpreter to complete the questionnaires. Women received a small honorarium upon completion of the surveys. Data collection averaged about 50 minutes per woman.

Five instruments were used to collect the data. *The Sociodemographic Characteristics of Women Survey* was used to determine sociodemographic characteristics. Age, marital status, education, years of residence in Canada and breast cancer-related variables were collected. Breast cancer-related variables include having a family history of breast cancer, performing or having BSE, having a CBE, having a mammogram, being educated about breast cancer and BSE, having heard about BSE, and accessing information about BSE. This survey included 25 items with nominal and binomial (Yes/No) answers.

*The Women’s Knowledge about Breast Cancer Survey* was used to collect breast cancer information (Kamińska et al., 2015; Sun et al., 2017). This survey comprises 19 items to evaluate women’s knowledge about breast cancer risk factors and screening methods. Knowledge scores ranged from 0–1 on each item (Vahabi, 2011). Each women’s overall baseline knowledge was assessed using her total score, which could range from 0 to 19. Scores ranging from 0 to 6 indicate low knowledge. Values ranging from 7 to 12 reflect a medium knowledge, while scores ranging from 13 to 19 show a high level of knowledge. Women’s scores showed a medium level of knowledge (M = 10.57, SD = 0.40).

*The Arab Culture-Specific Barriers to Breast Cancer Questionnaire (ACSB),* developed by Cohen and Azaiza (2008), was used to explore specific Arab cultural beliefs about breast cancer. The ACSB has been tested and validated with Sunni Muslims, Druze, and Christian Arab women in Israel and the Palestinian Authority (Cohen & Azaiza, 2008). The instrument comprises 21 items and five sub-scales (exposure barriers, social barriers, religious beliefs concerning cancer, environmental barriers, and uneasiness with own body). All items have five response choices ranging from strongly agree (1 point) to strongly disagree (5 points). A low score indicates a high level of cultural obstacles related to breast cancer screening behaviors. Cohen and Azaiza reported Cronbach’s alpha values ranging from 0.76 to 0.90. Our Cronbach’s alpha ranged from 0.70 to 0.87, demonstrating adequate reliability.

*The Cancer Stigma Scale (CASS)* developed by Marlow and Wardle (2014) measured cancer stigma in the non-patient population. The CASS comprises 25 items and six sub-scales (awkwardness, severity, avoidance, policy opposition, personal responsibility, and financial discrimination). These items measured women’s stigma. The items have six response choices ranging from disagree strongly (1 point) to agree strongly (6 points). A high score indicates a high level of stigma. Marlow and Wardle reported Cronbach’s alpha values ranging from 0.73 to 0.87. In our study, the Cronbach’s alpha ranged from 0.61 to 0.77, indicating acceptable reliability.

*The Revised Champion’s Health Belief Model Scale (*Champion, 1999*) (CHBMS)* is a validated and reliable scale used to assess: 1) susceptibility (women believe they have the chance of getting breast cancer) (5) items; 2) seriousness (the belief that breast cancer is a serious illness) (7) items; 3) benefits (perception of benefits from BSE) (6) items; 4) barriers (perceived barriers to having a BSE) (6) items; 5) confidence (women’s ability to take action) (11) items; 6) health motivation (women’s motivation to perform the health behaviours) (7) items; 7) benefits of mammogram (6) items and; 8) barriers to mammogram (perceived barriers to having mammogram) (5) items. The scale has been used among Qatari (Donnelly et al., 2013a), Turkish (Secginli & Nahcivan, 2006), Saudi (Abolfotouh et al., 2015), and Israeli Arab women (Soskolne et al., 2007). The revised version of the CHBMS was used to assess perceived barriers and benefits of breast health. The 53 items have five response choices ranging from strong disagreement (1 point) to strong agreement (5 points). Each item has a maximum score of five, with higher scores indicating stronger breast health perceptions. Champion (1999) reported Cronbach’s alpha values ranging from 0.69 to 0.83. In our study, the Cronbach’s alpha ranged from 0.59 to 0.89.

## Data analysis

Data were analyzed using SPSS® (SPSS Inc., Chicago, IL) version 25.0 for® Windows® (Microsoft Corporation, Redmond, WA). Descriptive statistics were calculated to determine the distribution of the women's sociodemographic characteristics, practices about breast health, knowledge of breast cancer screening methods and risk factors. We also used descriptive statistics to explore women’s health beliefs, cancer stigma, and Arab culture-specific breast cancer screening barriers. Pearson correlation tests were used to examine the relationships between women’s health beliefs about breast health and predictor variables (age, marital status, education, knowledge score about breast cancer risk factors and screening, cancer stigma and Arab culture-specific barriers of breast cancer screening).

Logistic regression analysis (Tabachnick & Fidell, 1996) tested relationships between performed BSE, attended CBE, had a mammogram and sub-scales’ scores. Categorical variables (age, education, marital status, has information about BSE) and numerical variables (knowledge score, subscales of cancer stigma, sub-scales of cultural barriers to cancer screening, sub-scales of Arab culture-specific barriers to breast cancer screening and subscales of health beliefs) were identified as covariates. A backward stepwise (conditional) regression method was used. A Wald-type chi-square test assessed the significance of each independent variable in the bivariate model. The level of statistical significance was set a 0.05 for all analyses.

## Results

Women’s mean age was 37.9 years (SD = 12.4). All women practiced the Islamic faith. Eighteen women came from a refugee camp. Forty-four women had lived in Canada between 1 to 3 years. Fifty-three women were married. Nearly 50% of the women had an elementary school education. Only 14 women had a technical college or university education. All participants were unemployed. Twenty-four women (32%) performed BSE, 9 women (12%) had a CBE, and 5 (6.7%) had mammography. Seventy-two (96%) women had heard about BSE. Almost 83% of women believed that BSE is necessary. The majority (96%) of women had heard of BSE, and 17 (22.7%) women had a family or relative history of breast cancer. Participants’ mean score on breast cancer risk factors and screening knowledge was at mid-point (Mean = 10.57, SD = 0.40) (Table 1).

**Table 1.** Socio-demographic characteristics of women and practices on BSE, CBE, and mammography.

Variables	Frequency (%)
Mean of age	37.9 ± 12.48 (Min 18, Max 65)
Was in a refugee camp	
Yes	18 (24.0)
Years of residence in Canada	
Less than 1 year	24 (32.0)
1–3 years	44 (58.7)
3–5 years	7 (9.3)
Marital status	
Married	53 (70.7)
Other (Single, Widowed, Divorced)	22 (29.3)
*Education*	
Elementary	37 (49.3)
High school	24 (32.0)
Technical College/University	14 (18.7)
Have a breast self-examination anytime during the lifetime (Yes)	24 (32.0)
Have a clinical breast examination anytime during the lifetime (Yes)	9 (12.0)
Have a mammogram anytime during the lifetime (Yes)	5 (6.7)
Heard about breast self-examination (Yes)	72 (96.0)
Source of information (n = 72)	
Radio	5 (6.9)
Public advertisement	9 (12.5)
Internet	30 (41.7)
Medical/health clinic	19 (26.4)
Friends	9 (12.5)
Have a family or relative history of breast cancer	17 (22.7)
Mean total knowledge about breast cancer risk factors and screening^a^	10.57 ± 0.40

^a^Knowledge score measured on 0–1 scale for each item, and the total for all items is 19.

## Perceived barriers and benefits

Table 2 illustrates the correlations between age, cancer stigma, and Arab culture-specific barriers to BSE scores and the Champion Health Beliefs Model's outcome variables. A high score on the Arab culture-specific barriers to BSE indicates a high level of perceived seriousness about breast cancer (r = .296, *p* < .05). In this study, we found that perceived barriers and perceived benefits of BSE were negatively correlated. As women’s perceived barriers to BSE increase, the perceived benefits of BSE decrease (r = −.545, *p* < .05). Results show that an increase in confidence correlates with reducing women’s susceptibility to breast health (r = −.296, *p* < .05). Health motivation is positively associated with the confidence of performing BSE (r = .242, *p* < .05). A similar trend exists between confidence and the likelihood of undergoing a mammogram (r = .245, *p* < .05). Syrian refugee women who have a positive perception of BSE coupled with a high level of motivation are likely to feel more confident about performing BSE. Perceptions of the benefits of BSE affect barriers to mammograms. The more positive women’s perceptions of the benefits of BSE, the less likely women will see barriers to undergo a mammogram (r = −.545, *p* < .01). In corollary, negative or low perceptions of the benefits of BSE strongly increase women’s barriers to mammograms (r = 1000*, p<.01*) (Table 2).

**Table 2.** Pearson correlation coefficients between predictor variables and health belief model.

	*M*	*SD*	1	2	3	4	5	6	7	8	9	10	11	12
Age (1)	37.94	12.41	1											
Knowledge (2)	10.57	3.54	–.019	1										
Cancer stigma (3)	1.99	0.65	–.049	–.069	1									
ACBS (4)	2.73	0.70	.035	–.157	.137	1								
Susceptibility (5)	12.17	3.61	218	–.196	.088	–.086	1							
Seriousness (6)	22.88	4.79	–.030	–.117	.005	.296*	.148	1						
Benefits of BSE (7)	14.85	3.13	–.276*	–.150	.212	.174	–.078	.187	1					
Barrier to BSE (8)	12.96	3.59	–.055	–.220	.139	–.004	–.048	–.009	.–545*	1				
Confidence (9)	29.97	7.06	–.118	–.049	.163	.100	–.296*	–.022	–.168	–.099	1			
Health mot. (10)	27.34	4.03	–.118	.048	.053	.220	–.026	.207	–.089	–.037	.242*	1		
Benefits of mam. (11)	22.80	3.54	–.153	.140	–.124	–.064	–.128	.093	–.071	–.191	.245*	–.061	1	
Barriers to mam. (12)	12.96	3.59	–.055	–.220	.139	–.004	–.048	–.009	–.545**	1000**	–.099	–.037	–.191	1

*0.05 (2-tailed)

**0.01 (2-tailed)

## Predictors of BSE, CBE, and mammography

The predictors of women on BSE, CBE and mammography are presented in Table 3. Knowledge, having information about BSE (risks), policy opposition, responsibility, barriers to BSE, and seriousness statistically significantly *(Cox & Snell R^2^ = .23, −2 log-likelihood = 77.320, X^2^ = 13.652; df = 1, p < .01)* predict refugee women’s BSE practices. Women were more likely to perform BSE if they knew about breast cancer screening and risk factors (OR = 1.25, CI = 0.98–1.59), had information about BSE (OR = 5.41, CI = 1.35–21.70), believed in the seriousness of BSE (OR = 1.30, CI = .96–1.70), and responsibility (OR = 38.11, CI = 11.57–92.73). Policy opposition and barriers to BSE strongly predict low BSE uptake and performance. As the scores of policy opposition (e.g., government priorities on breast cancer) (OR = 0.14, CI = 0.02–0.90) and barriers to BSE (OR = 0.61, CI = 0.38 –0.96) increase, the tendency to perform BSE decreases.

**Table 3.** Predictors of breast cancer screening practices.

	B (S.E.)	*p*	Wald	OR	95% CI	–2 Log likelihood	Cox & Snell *R*^2^
BSE							
Knowledge level	1.57 ( .28)	0.04*	3.967	1.25	0.98–1.59	77.320	.239
Have information BSE	2.70 (1.23)	0.02*	4.799	5.41	1.35–21.17		
*Cancer stigma*							
Policy opposition	–1.93 ( .93)	0.03*	4.290	.14	0.023–0.90		
Responsibility	3.64 (1.62)	0.02*	5.013	38.11	1.57–92.73		
*Health beliefs*							
Barrier to BSE	–.49 (.23)	0.03*	4.510	.61	0.38–0.96		
Seriousness	.26 (.13)	0.05*	3.724	1.30	99–1.70		
*p < 0.05							
CBE							
*Arab culture barriers*						69.067	.234
Religious beliefs	–1.28 (.71)	0.04*	3.198	.27	0.068–1.13		
*Health beliefs*							
Benefit of BSE	–.46 (.26)	0.07**	3.126	1.58	0.95–2.64		
*p < 0.05, **p < 0.10							
Mammography							
Education	6.47 (3.13)	0.03*	4.254	58.97	.69–498.03	64.614	.337
Age	5.48 (2.66)	0.04*	4.223	239.73	1.28–446.93		
Knowledge score	.65 (.331)	0.04*	3.909	1.92	1.00–3.67		
*Cancer stigma*							
Responsibility	5.30 (2.703)	0.05*	3.849	200.86	1.00–401.12		
*Health beliefs*							
Susceptibility	.94 (.503)	0.05*	3.552	2.58	0.96–6.92		
*Arab culture barriers*							
Social barrier	3.04 (1.582)	0.05*	3.699	20.96	944–465.71		
Religious beliefs	–6.66 (3.939)	0.06**	2.868	.00	0.00–2.85		
*p < 0.05, **p < 0.10							

CI: confidence interval; OR: odds ratio; S.E.: standard error.

Bivariate logistic regression. *P* value indicates significance from a bivariate regression analysis.

Benefits of BSE and religious beliefs were found to be statistically significant *(Cox & Snell R^2^ = .23, -2 log-likelihood = 69.067, X^2^ = 12.480; df = 1, p < .01)* in predicting whether women would undergo a clinical breast examination. Women were more likely to have a CBE if they had low religious belief scores (OR = 0.277, CI = .068– 1.13). Also, when the benefits of BSE increase (OR = 0.610, CI = .386 - .963), women's odds to use CBE decrease.

Age, education level, knowledge score, responsibility, susceptibility, social barriers and religious beliefs were statistically significant in women’s mammography use *(Cox & Snell R*^2^ = .33, −2 log-likelihood = 64.614, *X*^2^ = 19.095; *df* = 1, *p* < .01). Women with strong religious beliefs (OR = 0.001; CI = .000– 2.85) were less likely to have a mammogram (Table 3). Women were more likely to have a mammography if they were more educated (OR = 58.97, CI = 0.69– 498.03) and knew about breast cancer screening and risk factors (OR = 1.92, CI = 1.00–3.67). Age was a significant predictor of mammography. Women aged 40–65 were more likely to have a mammogram than younger women aged 18–39 (OR = 239.73, CI = 1.28 – 446.93). Women were more likely to have a mammogram if they had responsibility (OR = 200.86, CI = 1.00–401.12), susceptibility to breast health (OR = 2.58 CI = .96–6.92) and fewer social barriers to access and use screening (OR = 20.96, CI = 0.94– 465.71). Responsibility refers to looking after young children and spouses.

## Discussion

Breast cancer screening represents a concern among women from ethnocultural minorities and specifically among non-Western refugee women. In Canada and other Western countries, refugee women have lower screening and higher mortality rates related to breast cancer screenings (Ginsburg et al., 2015; Lofters et al., 2019; Vahabi, 2011). Over the past years, Canada accepted many refugees from the Middle East, Iraq, Somalia, and Ethiopia (Government of Canada, 2020). The literature review indicates that Muslim women and Muslim refugee women may present cultural beliefs that influence perceptions of cancer and breast health-seeking behaviors. Yet, evidence remains scarce in Canada and Western countries. Our study addresses this gap as our team explored how knowledge, cancer stigma, and Arab culture influence Muslim refugee women’s breast screening practices.

## Women’s knowledge about breast cancer and screening

In our study, refugee women present an average level of knowledge about BSE and breast cancer screening practices like clinical breast examination and mammograms. Mamdouh et al. (2014) found that Egyptian women had poor knowledge of breast cancer, while Petro-Nustas et al. (2012) also reported that Arab women had insufficient knowledge. Qatari and Israeli studies showed that many Arab women presented low level or limited knowledge about breast cancer and screening methods (Donnelly et al., 2013a; Soskolne et al., 2007). In parallel, Muslim women who live in Canada and the United States of America had limited knowledge of breast cancer and screening methods (Raymond et al., 2014; Saadi et al., 2012, 2015; Vahabi, 2011; Zorogastua et al., 2017). Our finding is not consistent with previous studies’ results and could be explained by the fact that most of our participants had heard about BSE. In contrast, some others (22.7%) had a relative history of breast cancer in Syria.

In our sample, a minority of women had a BSE, CBE, and mammograms, even if nearly 96% of them had heard about BSE. This low participation may be due to a lack of knowledge about the health care system and communication barriers that may affect access to a health care provider. Cultural barriers, gender roles, stigma, fear of losing one’s social status may also influence access to breast cancer knowledge, self-care (BSE), and other preventive measures. In their study among Arabic women in Australia, Kwok et al. (2016) found that only 7.6% practiced breast self-examination, 21.4% had undergone clinical breast examination, and 40.3% had biannual mammography. Mamdouh et al.’s (2014) report that Egyptian women had poor knowledge of breast cancer. Qatari women had a low level or limited knowledge about breast cancer and screening methods (Donnelly et al., 2013a). Muslim refugee women living in Canada (Vahabi et al., 2015, 2016) and the U.S. (Alatrash, 2020; Islam et al., 2017; Petro-Nustas et al., 2012; Saadi et al., 2015; Salman, 2012) have limited knowledge of breast cancer and low exposure to early screening practices. In our study, women’s low participation rate in breast screening practices may be explained by a lack of education and fluency in English or French, impeding the search and the understanding of health information.

Many of our participants used the internet and health providers as sources of information. In contrast, women seldom used the radio, public advertisement, and friends to gain knowledge. Women were not fluent in English as almost all Syrian refugee women spoke only Arabic while others were illiterate in their native language. Lack of fluency may explain the lack of use of mainstream media. Some researchers reported participants finding information about breast cancer and screening practices from various sources, mainly from physicians and newspapers (Donnelly et al., 2013a; Mamdouh et al., 2014). The literature review indicates that participation in breast cancer screening is low among Muslim women either in their home countries or abroad (Donnelly et al. 2013b; Islam et al., 2017; Petro-Nustas et al., 2012; Salman, 2012; Secginli & Nahcivan, 2006; Soskolne et al., 2007, Vahabi, 2011; Vahabi et al., 2015; Zorogastua et al., 2017). Despite knowing about breast cancer and BCE, women in our sample appeared to be unaware of the importance of breast cancer screening methods.

## Arab-culture specific barriers

Our study indicate that Arab culture-specific barriers like religious beliefs were a common barrier to breast cancer health. We found a positive correlation between Arab culture-specific barriers and women’s perceived seriousness of breast cancer. Women were more likely to perceive breast cancer as a severe illness if they had strong religious beliefs in the value of protecting their health. Protecting one’s health relates to Arab Culture-Specific Barriers’ domain of religious beliefs concerning cancer (Cohen & Azaiza, 2008). However, this increased seriousness did not translate into doing BSE or accessing CBE and mammograms. Cohen and Azaiza (2008) underlined that Islamic religious might increase Muslim women’s BSE practice but do not increase CBE participation and mammograms. Nyblade et al. (2017) reported that cancer stigma might lead to the fear of casual transmission of cancer, personal responsibility for having caused cancer, and fear of the inevitability of disability and death with a cancer diagnosis. Breast cancer has the potential to create disharmony and imbalance in the lives of people and their relatives and friends (Flynn & Ormseth, 2011). Some factors such as culture, religion, stigma, and health beliefs can affect cancer screening among women and delay diagnosis and treatment (Cohen & Azaiza, 2010; Hatefnia et al., 2010; Islam et al., 2017; Zorogastua et al., 2017). We suggest that partnering with the communities can decrease some of the barriers related to Arab culture-specific barriers like religious beliefs. Our results reveal the need to educate women on the importance of early detection of cancer, especially as they arrive in a host country where screening is physically and financially accessible.

## Predictors of breast self-examination (BSE)

Our results indicate that Muslim refugee women were more likely to perform BSE if they knew about breast cancer screening and risk factors. They were also more likely to perform BSE if they believed in the seriousness of breast cancer, the usefulness of BSE, and felt responsible for their health (e.g., remaining healthy to look after their families). Secginli and Nahcivan (2006) reported that low barriers to BSE are significantly associated with the performance of BSE among Turkish women. Shiryazdi et al. (2014) found that perceived seriousness was not a significant predictor of BSE performance among Iranian women. These conflicting results deserve further exploration as these two studies were conducted with non-refugee women in Turkey (Secginli & Nahcivan, 2006) and Iran (Shiryazdi et al., 2014). Besides, our findings suggest that being knowledgeable about breast cancer and its risk factors, having information about BSE, low policy opposition, and decreased cancer stigma significantly predict BSE practice. Low policy opposition occurs when governments or agencies support cancer care, facilitate cancer treatment, and prioritize cancer patients’ access to health care. Low opposition enables the performance of BSE. To our knowledge, limited studies have explicitly examined the association between policy opposition and breast cancer screening uptake. In a study carried in the United Kingdom, Vrinten et al. (2016) reported that low scores on policy opposition among ethnocultural minorities’ women facilitate cervical, breast, and colorectal cancer screening uptake. The Canadian health care system covers physician consultations, hospitalizations, and treatments, translating into low policy opposition (Sutcliffe, 2011). Therefore financial cost does not represent a barrier to breast cancer screening and treatment. Our study shows that Muslim refugee women lack knowledge of the Canadian health system, had little knowledge about breast health, and did not know how to access breast cancer screening. Interventions must be developed to increase Muslim refugee women’s knowledge about the health care system and access breast cancer screening in Canada. Comparative studies between refugee host countries’ public and private health care systems can further explore the impact of policy opposition on Muslim refugee women’s access to BSE information and the use of breast screening methods.

## Predictors of clinical breast examination (CBE)

Our results indicate that Muslim refugee women were more likely to have a CBE if they were less adherent to religious beliefs. Previous studies showed that religion could act as a facilitator to breast cancer screening (Islam et al., 2017; Padela et al., 2015, 2018; Raymond et al., 2014). Azaiza et al. (2010) found that a high CBE rate was related to being less religious. They also reported that being less religious was associated with a higher level of perceived effectiveness of CBE and lower cancer fatalism. Gender exposure barriers (physician gender), cultural barriers (modesty), social barriers (loss of status among family/friends) and environmental barriers (lack of financial and geographic access to care and lack of fluency in the host country’s language) negatively impacted Muslim women’s CBE practices (Cohen & Azaiza, 2010). It is not surprising that those who believe in self-examination benefits are far from performing a clinical breast examination as CBE requires consultation with health care providers. The gender of the health care provider may influence women’s decision to undergo a CBE as Muslim women, as less likely to expose their breasts to male health care providers (Alatrash, 2020). Islamic faith may influence gender relations and health-seeking behaviors. Gender may add complexities to professional health care encounters (Alatrash; Kerner et al., 2015) as women with strong religious beliefs may avoid breast clinical breast examinations. It will be necessary to explore how gender may affect Muslim refugee women’s use of clinical breast examination services in Canada and delineate interventions to decrease gender barriers.

## Predictors of mammography

Our findings indicate that Muslim refugee women who are more educated and know breast cancer screening and risk factors are more likely to have a mammography. Women aged 40–65 were more likely to have a mammogram than those aged 18–39. Age is an important predictor of mammography. Perceived susceptibility, perceived benefits, low social and religious barriers positively predict BSE and mammograms. Muslim refugee women who perform BSE are more likely to undergo mammograms. Donnelly et al. (2013a) and Ahmed et al. (2015) also found that greater knowledge of breast cancer and screenings were positively correlated with higher screening rates.

Similarly, Zorogastua et al. (2017) indicated that a lack of knowledge and misconceptions on breast cancer increase barriers to women’s cancer screening practices. In parallel, one study reported that increased perceptions of seriousness of breast cancer and positive perceptions about mammography benefits were significantly associated with having a mammogram (Secginli & Nahcivan, 2006). Previous results underlined the influence of religious and cultural differences in lower screening participation (Islam et al., 2017; Padela et al., 2015). As such, Padela et al.’s (2018) suggest using Muslim faith-tailored interventions to increase Muslim U.S. women’s mammography rates. Previous Canadian researchers (Vahabi, 2011; Vahabi et al., 2015, 2016) reported lower breast screening rates among Muslim immigrant women. This finding aligns with our results among a group of Muslim refugee women. More studies on recent Muslim refugee women will need to be conducted to contextualize and expand our findings.

Our study presents some limitations. The major limitation resides in the use of a sample of convenience. The findings cannot be generalized to all Muslim refugee women in Canada or globally. We cannot apply the results to highly educated immigrant and non-immigrant Muslim women as our sample was composed of women refugees with elementary education. We suggest replicating this study with larger samples of Muslim refugee women to increase generalizability. We recommend exploring breast cancer and stigma primarily within the context of Muslim refugees’ resettlement in Canada and globally. The impact of acculturation on Muslim refugee women’s breast cancer practices also needs further examination through qualitative and mixed- methods longitudinal designs. Our study provides valuable insights to understanding breast screening barriers and facilitators among these women born in Syria and residing in Canada and supporting the ongoing need for screening programs to engage with Muslim refugee communities.

## Conclusion

Syrian refugee women share similarities in breast screening practices with Muslim women living in their home countries. Yet Muslim refugees present vulnerabilities that put them at a higher risk of avoiding early breast screening practices like BSE, CBE, and mammograms. There is an urgent need to educate Syrian and Muslim refugee women upon arrival in asylum countries to maximize health outcomes. Interventions should increase knowledge, confidence, and motivation to perform breast self-examination and undergo clinical breast examination and mammograms. The development of religious-based interventions is recommended to decrease Arab culture-specific barriers and improve breast cancer awareness, knowledge, and self-care practices. Ideally, interventions should be directed at younger women as they are more at risk of stigma, and they need to learn why and how to perform BSE.
